# Supplementary figures and images for: Publication delays and associated factors in ophthalmology journals
Source: PeerJ. 2022 Nov 3;10:e14331. doi: 10.7717/peerj.14331 (PMC9637357; doi:10.7717/peerj.14331)

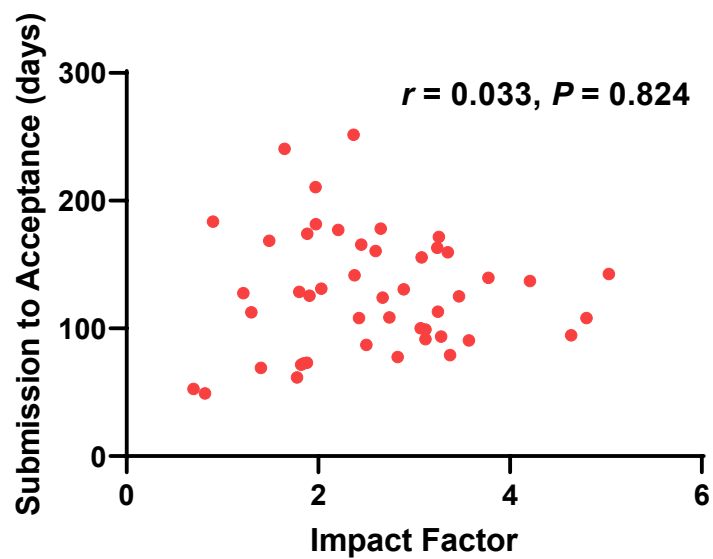

A

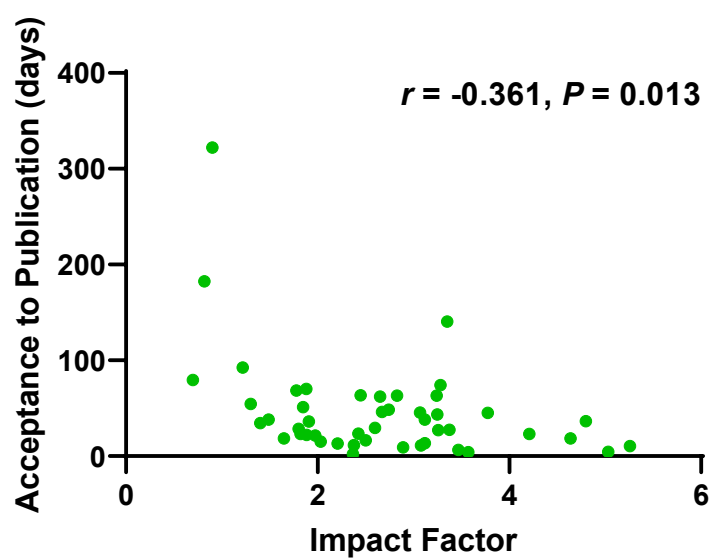

B

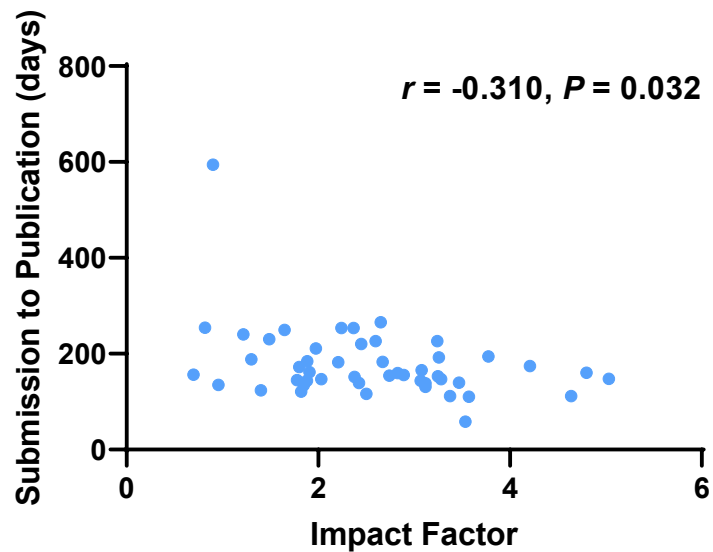

C

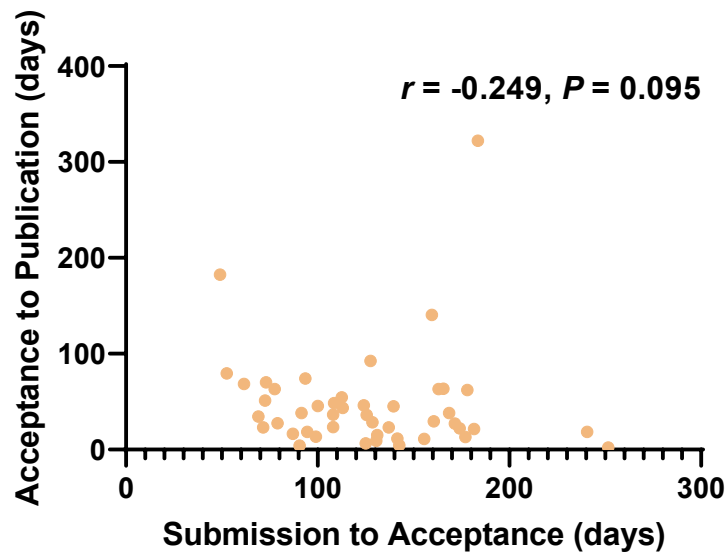

D

Supplement: Supplemental Information 1 — Scatterplots showing correlation statistics after two journals with the highest impact factors were excluded. (A) Correlation between the impact factor and SA (r = −0.033, P = 0.824), (B) Correlation between the impact factor and AP (r = −0.361, P = 0.013), (C) Correlation between the impact factor and SP (r = −0.310, P = 0.032). (D) Correlation between SA and AP (r = −0.249, P = 0.095). [file peerj-10-14331-s001.pdf]
