# Supplementary material for: Publication delays and associated factors in ophthalmology journals
Source: PeerJ. 2022 Nov 3;10:e14331. doi: 10.7717/peerj.14331 (PMC9637357; doi:10.7717/peerj.14331)
Supplement: Supplemental Information 2 — Availability of online advance publication and print publication is indicated. [file peerj-10-14331-s002.docx]

Supplemental Table 1: List of ophthalmology journals in the year 2020 with information on submission, revision and acceptance dates of the manuscripts. Availability of online advance publication and print publication is indicated.

| **Journal** | **Date of submission reported** | **Date of revision reported** | **Date of acceptance reported** | **Online advance available** | **Publication in print available** |
| --- | --- | --- | --- | --- | --- |
| Acta Ophthalmol | Y | P | Y | Y | Y |
| Am J Ophthalmol | N | N | Y | Y | NA |
| Arq Bras Ofthalmol | Y | N | Y | Y | NA |
| Asia-Pac Journal of Ophthalmol | Y | N | Y | P | NA |
| Br J Ophthalmol | Y | Y | Y | Y | Y |
| BMC Ophthalmol | Y | N | Y | N | Y |
| Can J Ophthalmol | Y | Y | Y | Y | NA |
| Clin Exp Ophthalmol | Y | Y | Y | Y | NA |
| Clin Exp Optom | Y | Y | Y | Y | NA |
| Contact Lens Anterior Eye | Y | Y | Y | Y | NA |
| Cornea | Y | Y | Y | Y | NA |
| Curr Eye Res | Y | N | Y | Y | NA |
| Cutan Ocul Toxicol | Y | N | Y | Y | NA |
| Doc Ophthalmol | Y | N | Y | Y | NA |
| Eur J Ophthalmol | Y | N | Y | Y | Y |
| Exp Eye Res | Y | Y | Y | Y | NA |
| Eye | Y | Y | Y | Y | NA |
| Eye Vis | Y | N | Y | N | Y |
| Eye Contact Lens-Sci Clin Pra | N | N | Y | P | NA |
| Graef Arch Clin Exp Ophthalmol | Y | Y | Y | Y | NA |
| Indian J Ophthalmol | Y | Y | Y | N | Y |
| Int J Ophthalmol | Y | Y | Y | N | Y |
| Invest Ophthalmol Vis Sci | Y | N | Y | N | Y |
| Int Ophthalmol | Y | N | Y | Y | NA |
| J AAPOS | Y | N | Y | Y | NA |
| JAMA Opthalmol | N | N | Y | Y | Y |
| J Cataract Refract Surg | Y | Y | Y | N | NA |
| J Eye Mov Res | Y | N | N | N | Y |
| J Fr Ophthalmol | Y | P | Y | Y | NA |
| J Glaucoma | Y | N | Y | Y | NA |
| J Neuro-Ophthal | N | N | N | NA | NA |
| J Ocular Pharmacol Ther | Y | N | Y | Y | Y |
| J Ophthalmol | Y | P | Y | N | Y |
| J Pediatr Ophthalmol Strabismus | Y | N | Y | Y | Y |
| J Refractive Surg | Y | N | Y | Y | Y |
| J Vision | Y | N | N | N | Y |
| Jpn J Ophthalmol | Y | N | Y | Y | NA |
| Klinische Monatsblat Augenheilkunde | Y | N | Y | Y | NA |
| Mol Vis | Y | N | Y | N | Y |
| Ocul Immunol Inflamm | Y | N | Y | Y | NA |
| Ocul Surf | Y | Y | Y | Y | NA |
| Ophthalmic Epidemiol | Y | Y | Y | Y | NA |
| Ophthalmic Physiol Opt | Y | P | Y | Y | NA |
| Ophthalmic Plast Reconstr Surg | N | N | Y | NA | NA |
| Ophthalmic Surg Lasers Imaging | Y | P | Y | Y | Y |
| Ophthalmic Genet | Y | Y | Y | Y | NA |
| Ophthalmic Res | Y | N | Y | Y | NA |
| Ophthalmologe | N | N | N | Y | NA |
| Ophthalmologica | Y | N | Y | Y | NA |
| Ophthalmology | Y | Y | Y | Y | Y |
| OPHTHALMOL THER | Y | N | N | Y | NA |
| Optom Vis Sci | Y | N | Y | NA | NA |
| Perception | Y | N | Y | Y | Y |
| Retin-J Retin Vitr Dis | N | N | N | NA | NA |
| Semin Ophthalmol | Y | P | Y | Y | NA |
| Transl Vis Sci Technol | Y | N | Y | N | Y |
| Vision Res | Y | Y | Y | Y | Y |
| Visual Neurosci | Y | Y | Y | N | Y |

Abbreviations: Y: yes, N: no; P: partial; NA: not available
